# The evolution and future trajectory of public hospital reforms with a public-welfare orientation in China

**DOI:** 10.3389/fpubh.2025.1723525

**Published:** 2026-01-15

**Authors:** Cheng Zhang, Linmi Deng, Yongzhulacuo, Qiuyu Pan

**Affiliations:** 1 Sichuan Health Development Research Center, Chengdu, China; 2 Guilin Medical University, Guilin, China; 3 Tibet University, Lhasa, China; 4 North Sichuan Medical College, Nanchong, China

**Keywords:** collaborative governance, development trajectory, public hospitals, public-welfare, reform process

## Abstract

As a cornerstone of the healthcare system, public hospital reforms in China represent a complex and critical endeavor, shaped by the nation’s evolving socioeconomic landscape. This paper employs a methodological framework of historical and policy analysis to examine the evolution of public hospital reforms in China. It traces the trajectory of these reforms against the backdrop of the country’s socioeconomic development across different periods and identifies four distinct stages: (1) the government-led system foundation stage (1949–1978), (2) the market-oriented phase marked by a gradual weakening of public-welfare nature (1979–2004), (3) the period of reflection and readjustment toward restoring public-welfare orientation (2005–2020), (4) the ongoing stage of deepening reform centered on reinforcing public-welfare nature (2021–present). A key contribution of this study lies in its integration of historical and policy perspectives, addressing a significant gap in the comparative literature on health system reforms. The analysis concludes that upholding public-welfare nature as the core principle is essential for evaluating the success of future reforms. To address existing policy barriers, the study proposes establishing a collaborative governance mechanism and strengthening the “tripartite linkage” among medical services, health insurance, and pharmaceuticals, coupled with a reconstruction of incentive systems to better align with public-welfare objectives. These insights offer valuable lessons for other developing countries pursuing similar health system transformations under complex socioeconomic conditions.

## 1 Introduction

President Xi Jinping has emphasized that “the fundamental positioning of public hospitals must uphold their public-welfare nature” (1). This position was echoed at the 20th National Congress of the Communist Party of China (CPC) and the Third Plenary Session of its 20th Central Committee, which explicitly called for “deepening public hospital reforms with a public-welfare orientation.” Further, the 2025 National Health Work Conference has outlined specific measures to advance this agenda (2). The World Health Organization (WHO) emphasizes placing public welfare and equity at the heart of health systems, achieving this through primary healthcare and system integration, now actively championing a “whole-of-government” and even “whole-of-society” collaborative approach, which resonates strongly with China’s “tripartite linkage” mechanism integrating medical services, health insurance, and pharmaceutical policies (3, 4).

Yet despite high-level policy consensus, the public-welfare mandate remains contested on the ground. Currently, public hospitals in China face dual challenges in realizing the public-welfare nature: externally, insufficient government fiscal investment and fragmented inter-departmental coordination impede the refinement of pricing and compensation mechanisms, in the absence of sufficient and sustainable government fiscal input, policies requiring hospitals to provide a large volume of low-cost services with public-welfare attributes essentially constitute a form of value extraction from these institutions. in turn, compels hospitals to seek compensation through alternative channels such as over-provision of medical services (5). Internally, limited improvements in service quality and operational efficiency constrain institutional performance (6–8), the pursuit of equity in service accessibility may come at the cost of individual choice and patient experience (9). This paper systematically reviews the historical evolution of public hospital reforms in China, framed within shifting socio-economic conditions and national health system reform values. This review therefore addresses the following central question: How has the “public-welfare nature” of public hospitals in China been defined, contested, and transformed across different socio-economic and policy eras, and what lessons can be drawn from this evolution to address the current implementation gaps?

## 2 Conceptualizing the public-welfare nature of public hospitals in China

While there is no single, standardized definition of “public-welfare nature” (gong yi xing) for public hospitals in national policy, its core principles are reflected in key legislative and policy documents. Academic interpretations also vary. Scholars have approached the concept from multiple perspectives.

Some define it through institutional functions. For instance, Lei (10) posits that the public-welfare nature of public hospitals refers to the extent to which residents in a given region can universally access and benefit from public hospital services. Li (11) argues that it reflects the alignment between hospital operational objectives and governmental will, as well as the goal of maximizing social welfare. More recently, Tang et al. (12) emphasize that public hospitals, benefiting from fiscal subsidies and tax exemptions, should deliver a suite of public-welfare nature services that meet basic societal health needs and align with governmental objectives. Luo (13) defines the public welfare of public hospitals from the perspective of public administration, suggesting that the public welfare of public hospitals refers to the positive externalities brought by medical products and services. Zheng Anqi et al. emphasize that the connotation of public welfare is, firstly, ‘non-profit’ and, secondly, ‘aimed at promoting public well-being (14). Others focus on service delivery, asserting that public hospitals must safeguard citizens’ fundamental right to health by ensuring fairness, appropriateness, and accessibility in care provision, while also maintaining efficiency and quality (15–17). (Table 1)

**Table 1:** Key dimensions of “Public-welfare nature” in public hospitals.

Dimension	Core question	Scholarly and policy emphasis
Equity and accessibility	To what extent can all residents, regardless of background, access and benefit from services?	Universal access; benefiting from services; fairness and appropriateness in care provision (10, 16).
Functional alignment	How well do hospital operations align with state objectives and social welfare maximization?	Alignment with governmental will; goal of maximizing social welfare (11, 17).
Quality and efficiency	How are fairness and accessibility balanced with the need for effective and sustainable service delivery?	Maintaining efficiency and quality in care provision while ensuring accessibility (15).

## 3 Historical evolution of public-welfare nature-oriented reforms in China’s public hospitals

### 3.1 Government-led institutional foundation (1949–1978)

#### 3.1.1 Driving forces and policy actors

This phase was fundamentally driven by the state’s need to consolidate legitimacy and support socialist industrialization through a welfare-oriented model. The central government, particularly the Ministry of Health, served as the primary actor, implementing directives to establish a nationwide healthcare network, by 1952, over 90% of counties had established health institutions (18). In rural areas, collective agricultural units (“production brigades”) became key local actors in operationalizing the “barefoot doctor” system (19).

#### 3.1.2 Governance mechanism

A state-corporatist model was adopted, characterized by centralized financial control (“unified revenue and expenditure”) and administrative rigidity, hospitals operated under centralized financial models such as “unified revenue and expenditure,” “full budget management with deficit subsidies,” and “itemized subsidies with budget caps” (20). The government functioned as both planner and provider, utilizing price controls and full subsidization of losses to enforce equity. This approach effectively mobilized limited resources but created a dependency culture among public hospitals, with efficiency and innovation largely neglected (21).

#### 3.1.3 Phase transition analysis

The shift away from this model was triggered by the systemic inefficiencies of the planned economy. As fiscal burdens grew unsustainable amid China’s economic reorientation, the rigid, state-dominated framework became a candidate for reform—setting the stage for the experimental market-oriented phase that followed (22) (Table 2).

**Table 2:** Key policy documents during the government-led foundation phase (1949–1978).

Issuing authority	Issuance year	Document title	Main content
Ministry of Health	1951	Decision on Strengthening and Developing Grassroots Health Organizations	Establish a three-tier health network covering urban and rural areas (county, township, village); implement “unified revenue and expenditure” financial management.
Central People’s Government	1951	Regulations of the People’s Republic of China on Labor Insurance	Establish social insurance systems for workers (pension, medical, work injury, maternity), ensuring basic health protection.
Central People’s Government	1952	Directive on Implementing Public Medical Care for State Employees	Provide medical care and preventive services to staff of government agencies, political parties, mass organizations, and their affiliated institutions.
Ministry of Health	1953	Decision on Establishing Health and Epidemic Prevention and Control Systems	Strengthen the construction of epidemic disease prevention and control systems at provincial, municipal, county, and district levels.
Ministry of Health and Ministry of Finance	1955	Joint Notice on Improving Hospital Financial Management	Implement “full budget management with deficit subsidies” financial model.

### 3.2 Market-oriented reforms and Erosion of public-welfare nature (1979–2004)

#### 3.2.1 Driving forces and policy actor

Aligned with national economic liberalization, this phase aimed to boost efficiency and reduce fiscal pressure. Key actors included reform-oriented ministries advocating for decentralization, local governments reducing healthcare subsidies, and hospital administrators eager to leverage new operational autonomy.

#### 3.2.2 Governance mechanisms

A decentralized, incentive-driven governance structure emerged, marked by management contracts (“five-fixed-one-reward”) and revenue retention policies. The state permitted drug markup and fee-for-service pricing to compensate for reduced subsidies—a form of regulated pseudo-commercialization. By the 1990s, drug revenue exceeded 50% of total hospital income (23, 24), fueling cost inflation. This led to profit-maximizing behaviors, distorting clinical incentives and weakening equity.

#### 3.2.3 Phase transition analysis

Widespread public dissatisfaction with “difficult and expensive healthcare,” (25) crystallized by the landmark 2005 State Council report declaring reforms “unsuccessful,” exposed the human and social costs of market excess. This crisis of legitimacy prompted a fundamental reorientation toward re-embedding public-welfare principles in the next phase. Economic reforms prompted the health sector to adopt market-inspired mechanisms. While intended to revitalize stagnant institutions, these reforms increasingly prioritized revenue generation over public service, weakening the public-welfare nature mandate (Table 3).

**Table 3:** Key policy documents during the market-oriented phase (1979–2004).

Issuing authority	Issuance date	Document title	Main content
Ministry of Health, etc.	1979	Opinions on Strengthening Hospital Economic Management Pilot Work	Launch pilot programs including “Five Fixed, One Reward” (fixed tasks, beds, staffing, technical indicators, financial subsidies, plus performance rewards) and implement “fixed subsidy, economic accounting, assessment and incentives.”
State Council	1981	Report on Resolving Hospital Losses	Officially permit adjustment of medical service prices to compensate for losses; initiate reform of unreasonable fee structures.
State Council	1985	Report on Several Policy Issues Concerning Health Reform [Guo Fa (1985) No. 62]	Advocate reform: relax policies, decentralize authority, mobilize multiple funding sources, broaden development paths for health services; gradually reform outdated fee systems.
State Council	1989	Opinions on Expanding Medical Services [Guo Fa (1989) No. 10]	Clarify pricing reform and market-based compensation policies; promote contract responsibility systems, paid part-time services, “using side businesses to support core services,” and “using industry to assist medicine.”
State Council	1992	Several Opinions on Deepening Health Reform	Define scope of state investment in public hospitals; call for reform of health administration, diversification of funding, transformation of operational mechanisms, and enhanced business development.
CPC Central Committee & State Council	1997	Decision on Health Reform and Development [Zhong Fa (1997) No. 3]	Aim to enhance vitality of the health sector, adapt to socialist market economy, follow internal laws, and establish a new system with strong macro-control and dynamic micro-operations.
State Council	2000	Guiding Opinions on Urban Healthcare System Reform [Guo Ban Fa (2000) No. 16]	Establish industry and institutional classification management; encourage hospital mergers, collaborations, and group formation; allow for-profit institutions to set prices autonomously and pay taxes.

### 3.3 Reflective realignment and public welfare restoration (2005–2020)

#### 3.3.1 Driving forces and policy actors

This phase was propelled by top-down political intervention to address equity failures and rebuild public trust. The State Council and later the National Healthcare Security Administration (established 2018) became powerful actors, steering systemic realignment through financing and regulation (26).

#### 3.3.2 Governance mechanisms

The state adopted a strategic purchaser model, deploying powerful new tools: Zero-markup drug policy and centralized procurement to dismantle the “drug supplementation” mechanism. Diagnosis-Intervention Packet (DIP) & Diagnosis-Related Group (DRG) payment reforms to align clinical incentives with cost-effectiveness (27). “Two permissions” salary reforms to decouple income from revenue volume.

#### 3.3.3 Phase transition analysis

Once the new systems were institutionalized, policy focus shifted from dismantling old structures to optimizing new ones. With basic access restored, public expectations evolved toward quality and responsiveness, motivating the current phase focused on “high-quality development.” (Table 4).

**Table 4:** Key policy documents during the reflective adjustment and public-welfare nature restoration phase (2005–2020).

Issuing authority	Issuance date	Document title	Main content
CPC Central Committee & State Council	2009	Opinions on Deepening the Reform of the Medical and Health System [Zhong Fa (2009) No. 6]; Notice on Implementing the Near-Term Key Plans for Healthcare Reform (2009–2011) [Guo Fa (2009) No. 12]	Establish four major systems (public health, medical services, medical insurance, drug supply) and reform eight mechanisms (management, operation, investment, pricing, supervision, innovation, talent, information, legal). Launch public hospital reform pilot programs.
Ministry of Health, etc.	2010	Guiding Opinions on Public Hospital Reform Pilot Programs [Wei Yi Guan Fa (2010) No. 20]	Uphold the public-welfare nature and leading role of public hospitals; outline nine key reform areas: service system, management system, governance mechanism, operational mechanism, compensation mechanism, regulatory mechanism, etc., to alleviate “difficulty and high cost in accessing care.”
General Office of the State Council	2012	Guiding Opinions on Pilot Reform of County-Level Public Hospitals [Guo Ban Fa (2012) No. 33]	Pilot in ~300 counties; focus on “four separations,” eliminate “drug-based compensation,” reform compensation mechanisms and autonomy; comprehensively reform management, compensation, personnel, pricing, payment, procurement, and supervision systems to establish a sustainable operational model (“maintain public-welfare nature, motivate staff, ensure sustainability”).
General Office of the State Council	2015	Implementation Opinions on Fully Launching Comprehensive Reform of County-Level Public Hospitals [Guo Ban Fa (2015) No. 33]	Require nationwide elimination of “drug-based compensation” in county hospitals by 2015; focus on management, operational mechanisms, price adjustment, personnel compensation, and medical insurance payment reforms.
General Office of the State Council	2015	Guiding Opinions on Pilot Reform of Urban Public Hospitals [Guo Ban Fa (2015) No. 38]	Eliminate profit-seeking mechanisms; clarify government responsibilities (leadership, guarantee, management, supervision); establish a new operational mechanism that “maintains public-welfare nature, motivates staff, and ensures sustainability”; expand pilot scope.
National Health and Family Planning Commission, etc.	2017	Notice on Fully Implementing Comprehensive Reform of Public Hospitals [Guo Wei Ti Gai Fa (2017) No. 22]	Deploy nationwide implementation of comprehensive public hospital reform, including full elimination of drug markups.
National Health and Family Planning Commission	2018	Notice on Consolidating Achievements in Eliminating Drug-Based Compensation and Continuously Deepening Public Hospital Reform [Guo Wei Ti Gai Fa (2018) No. 4]	Consolidate new compensation mechanisms; fully implement healthcare planning; improve modern hospital management; strengthen government investment; control unreasonable growth in medical costs; deepen reforms in key areas.

### 3.4 Deepening reform and elevating the public welfare mandate (2021–present)

#### 3.4.1 Driving forces and policy actors

The current phase responds to China’s “high-quality development” agenda, emphasizing governance modernization. Key actors include health regulatory bodies (e.g., National Health Commission) leveraging refined performance metrics, and Party committees enforcing political accountability (28, 29).

#### 3.4.2 Governance mechanisms

A mandatory performance governance system has emerged, where public-welfare indicators become hard constraints. The “one-vote veto” rule in hospital accreditation exemplifies this, making public welfare a non-negotiable prerequisite for organizational advancement (30). Reforms increasingly focus on systemic integration—deepening the “tripartite linkage” of medical services, insurance, and pharmaceuticals (31, 32).

#### 3.4.3 Theoretical integration

China’s pathway reflects a global search for hybrid health governance. It has moved from state hierarchy to market mimicry, then through re-regulation, toward an emergent model blending public stewardship with performance-based management—a significant experiment in sustaining equity within a complex, modernized health system (33) (Table 5).

**Table 5:** Key policy documents during the deepening reform phase (2021–present).

Issuing authority	Issuance date	Document title	Main content
General Office of the State Council	2021	Opinions on Promoting High-Quality Development of Public Hospitals [Guo Ban Fa (2021) No. 18]	Uphold people’s health as the center; strengthen the leading role of public hospitals; adhere to government leadership, public-welfare nature orientation, and public hospital dominance; promote three strategic shifts: development model (from scale expansion to quality-efficiency), operational mode (from extensive to refined management), and resource allocation (from material inputs to human and technological factors).
National Health Commission, etc.	2021	Notice on Implementing the Action Plan for High-Quality Development of Public Hospitals (2021–2025) [Guo Wei Yi Fa (2021) No. 27]	Raise the banner of public-welfare nature; build a high-quality, efficient, equitable, and coordinated public hospital system aligned with socioeconomic development and evolving health needs; strengthen primary care, regional collaboration, prevention-treatment integration, and traditional Chinese medicine.
CPC Central Committee	2022	Report Delivered at the 20th National Congress of the Communist Party of China	Emphasize “promoting Healthy China construction”; place people’s health in a priority strategic position; deepen healthcare reform; promote coordinated governance of medical insurance, medical services, and pharmaceuticals; deepen public-welfare nature-oriented public hospital reform; regulate private hospital development.
CPC Central Committee	2024	Decision on Further Comprehensively Deepening Reforms and Advancing Chinese Modernization	Propose “deepen healthcare reform”: deepen public-welfare nature-oriented public hospital reform; establish a service-based charging mechanism; improve compensation systems; implement dynamic staffing adjustment mechanisms.
National Health Commission, etc.	2025	Tertiary Hospital Accreditation Standards (2025 Edition) [Guo Wei Yi Zheng Fa (2025) No. 4]	List “public-welfare nature Responsibility and Professional Ethics” as Part I – “Prerequisite Requirements,” implementing a “one-vote veto” policy; require fulfillment of this criterion as a prerequisite for establishing key departments (e.g., pediatrics, infectious diseases).

## 4 Emerging trends in public-welfare-oriented reforms

### 4.1 Public-welfare nature as the core guiding principle

The public-welfare nature orientation of public hospitals has consistently served as the cornerstone and core value pursuit of China’s medical and health system reform. Retrospective analysis of the reform trajectory reveals that since the initiation of the New Healthcare Reform, the principle of “upholding the public-welfare nature of public hospitals” was institutionalized as the guiding ideology and foundational principle, profoundly shaping the reform’s top-level design and policy direction.

In the new era, this principle has attained unprecedented strategic prominence. The 20th National Congress of the Communist Party of China and the Third Plenary Session of the 20th Central Committee explicitly emphasized “deepening public hospital reforms oriented by public-welfare nature,” with the core imperative being the explicit requirement that all reform stages and policy frameworks must unequivocally embody and operationalize public-welfare nature principles, elevating them to a position of paramount importance (34). This represents both a concrete manifestation of the CPC Central Committee and State Council’s “people-centered” development philosophy in the health sector and a profound reclamation of the essential attributes and social functions of public hospitals (35).

Currently, as public hospital reforms enter a new historical phase, the “public-welfare nature orientation” has transcended its foundational role to become the central criterion for guiding reform trajectories and evaluating reform outcomes. This coincides with healthcare reforms in countries like the UK. The structural reform measures implemented by the NHS under the premise of maintaining public welfare have greatly promoted orderly competition among medical institutions and diversified development in the provision of medical services (36).

### 4.2 Interdepartmental collaborative governance as an enabler

The reform agenda outlined at the 2025 National Health Work Conference—including the establishment of a dynamic staffing quota adjustment mechanism, a service-oriented medical pricing system, improved compensation schemes, innovative healthcare regulatory approaches, and optimized fiscal subsidy policies—extends far beyond the capacity of health administrative departments acting alone. These reforms profoundly intersect with the mandates, policy instruments, and institutional boundaries of multiple core government agencies, including those responsible for human resources and staffing (e.g., the Office of the Central Institutional Organization Commission), finance, health insurance, and health. The resulting policy network is highly complex, with interwoven initiatives that simultaneously reinforce and constrain one another. Policy silos, divergent institutional objectives, and delayed inter-agency coordination can easily become critical bottlenecks that impede the realization of public-welfare nature-oriented reform goals.

Consequently, establishing an efficient and seamless cross-departmental collaborative governance mechanism—particularly by deepening and operationalizing the empirically validated “Trilateral Coordination” (san yi lian dong) framework (integrating medical services, health insurance, and pharmaceuticals)—is essential as both an institutional safeguard and a core driver for the successful implementation of public hospital reforms centered on public-welfare nature. Relevant departments must align their actions around this shared public-welfare nature objective, precisely leveraging their respective mandates to ensure policy coherence, synchronized implementation, and avoidance of contradictory measures or counterproductive outcomes, thereby maximizing the synergistic effects of the policy mix, health authorities oversee medical service quality supervision, medical insurance departments lead payment method reforms, and drug regulatory authorities manage the quality control of pharmaceuticals and medical consumables. For example, Sanming’s medical reform in China takes public welfare as the core of the healthcare system, and drives the coordinated development of the “Trilateral Coordination” (san yi lian dong) through three mechanisms: consensus-driven guidance, rule-based constraints, and organizational safeguards (37).

Moreover, public hospitals, as the pivotal nexus of the Trilateral Coordination framework, inherently demand more sophisticated health insurance payment models and refined management of centralized procurement and utilization of pharmaceuticals and medical consumables as their public-welfare nature reforms advance (38). Practical inter-departmental collaboration focused explicitly on public-welfare nature continuously reveals systemic bottlenecks and implementation challenges within the Trilateral Coordination chain, thereby generating empirical evidence and endogenous momentum for further refinement and deepening of the coordination mechanism itself. This dynamic fosters a virtuous cycle: enhanced inter-agency collaboration strengthens public-welfare nature, while the pursuit of public-welfare nature, in turn, reinforces and evolves the Trilateral Coordination framework.

### 4.3 Reconstructing incentive mechanisms to align motivation with mission

The root cause for the weakening of the public-welfare nature and problems such as “difficult and expensive access to healthcare” lies in a historical deficit in government financial compensation which, when compounded by early market-oriented reforms, created a systemic reliance on profit-seeking behaviors such as “drug markup compensation,” thereby undermining equity and accessibility. The key to restoring public-welfare nature lies in systematically reconstructing incentive mechanisms for medical staff, transforming their personal drive for value realization into a public-welfare nature-oriented motivation through effective institutional arrangements (39). For incentive mechanism reconstruction introduce quantitative standards such as trigger conditions for dynamic adjustments to medical service prices and public welfare indicators account for no less than 40% of performance-based salaries.

Specific pathways include: first, strengthening government responsibility by implementing the six types of fiscal subsidies, effectively utilizing policy loss subsidies, increasing the proportion of fiscal revenue, and establishing a solid foundation for public-welfare nature; second, deepening reforms in personnel and compensation systems by fully utilizing existing vacant positions, establishing dynamic staffing adjustment mechanisms, fully implementing the “two allowances” policy, progressively advancing the “three structural adjustments,” and reasonably utilizing cost savings from centralized procurement of drugs and medical supplies to dynamically adjust medical service prices, gradually increasing the proportion of medical service income, and formulating management measures aligned with the compensation system; third, improving mechanisms for medical professional risk prevention and dispute resolution to foster a favorable professional environment and societal atmosphere that respects and values healthcare professionals. Through the reconstruction of incentive mechanisms, the technical and labor value of medical staff can be fully reflected, enhancing their initiative in patient care, creativity in quality improvement, and sense of responsibility in cost control, thereby providing robust and sustainable internal support for the public-welfare nature objectives of public hospitals.

## 5 Summary

Thus China’s journey in defining and institutionalizing the public-welfare nature of its public hospitals offers a profound case study of navigating the enduring tensions between equity and efficiency, state and market. The analysis yields critical policy implications for future reform design. Success in the ongoing phase of deep systemic integration will hinge on unwavering political commitment to prioritize social welfare, robust inter-agency coordination particularly through the “tripartite linkage” of medical services, insurance, and pharmaceuticals to overcome fragmented governance, and the careful reconstruction of incentive structures that align hospital and physician behaviors with public-welfare objectives rather than purely revenue-driven metrics.

## 6 Limitations and future research

This analysis is primarily descriptive and conceptual, drawing on policy documentation and historical review. Its conclusions, while insightful for framing the reform landscape, lack quantitative validation. Besides insufficient analysis of regional heterogeneity and failure to consider the impact of digital transformation on public welfare; objectively illustrate constraints on data availability to enhance research rigor. Future research should empirically test the relationships proposed herein, for instance, by employing econometric models to measure the impact of specific policy interventions on public-welfare outcomes or by conducting comparative case studies across provinces to identify the contextual factors that determine the success or failure of different reform models. Research on the collaborative mechanism of public welfare between primary public hospitals and tertiary public hospitals and specify research methods to provide clear guidance for subsequent studies.

Although these limitations, the Chinese experience contributes significantly to global debates on health system reform and governance. It demonstrates that upholding public-welfare principles in a complex, modernizing economy is not about a simple return to a planned model, but rather about constructing a sophisticated hybrid governance framework. This framework must intelligently blend state steering with calibrated market mechanisms, offering a replicable analytical lens for other nations, particularly emerging economies, striving to build health systems that are both equitable and sustainable in the long run.
